# Mycoplasma pneumonia with severe cold agglutinin hemolysis, thrombocytosis, leukemoid reaction and acute renal failure

**DOI:** 10.1016/j.idcr.2023.e01689

**Published:** 2023-01-13

**Authors:** Johan Widén, Göran Jönsson, Ulf Karlsson

**Affiliations:** a Department of Infectious Diseases, Skåne University Hospital, Lund, Hälsogatan 3, 221 85 Lund, Sweden; b Department of Clinical Sciences, Section for Infection Medicine, Lund University, Lund, Sweden

**Keywords:** Mycoplasma pneumoniae, Cold-agglutinin hemolysis, Acute kidney failure, Leukemoid reaction, Corticosteroids

## Abstract

*Mycoplasma pneumoniae (M. pneumoniae)* is a common cause of community acquired pneumonia and although most cases are mild, complications sometimes occur. Cold agglutinin hemolysis is a known complication of *M. pneumoniae* infection, and usually presents as a mild and transient hemolysis. Here we present a case of infection with *M. pneumoniae* in a 64-year-old male that caused life threatening hemolysis that required multiple blood transfusions. The patient also presented with acute kidney failure and a marked leukemoid reaction and thrombocytosis. This is a very rare combination of symptoms that could have led the clinicians to suspect a more virulent etiology than *M. pneumoniae*, thereby delaying adequate antibiotic treatment.

## Highlights

• *Mycoplasma pneumoniae* caused life threatening cold agglutinin hemolysis.
• Treatment includes warm blood transfusions and keeping the patient warm.
• Glucocorticoids and adequate antibiotic treatment are likely beneficial.
• *M. pneumoniae* should be considered in pneumonia with hematological abnormalities.

## Introduction

*Mycoplasma pneumoniae* (*M. pneumoniae*) is a small rod-shaped bacterium without a cell wall. It is one of the most common causes of community-acquired pneumonia, especially in younger individuals. It generally causes a mild respiratory infection that rarely requires antimicrobial therapy, although severe pneumonia with respiratory failure requiring hospitalization occurs. In up to 25% of infected patients, *M. pneumoniae* infection can also cause extra-pulmonary manifestations such as skin rashes, encephalitis, myocarditis and hemolysis [1]. Renal complications such as acute glomerulonephritis and renal failure have been reported sporadically in association with *M. pneumoniae* infection and heterogeneous mechanisms of kidney injury have been suggested [2], [3], [4], [5], [6]. Here we present a case of *M. pneumoniae*-pneumonia that presented with severe hemolysis, acute kidney failure and a leukemoid reaction.

## Case report

The patient is a 64-year-old male with a medical history of mild chronic obstructive lung disease, requiring no treatment, and psoriasis treated with Methotrexate. He presented at the emergency department with a 10-day history of fever and dry cough. Three days prior to admission he had been prescribed Amoxicillin, without clinical improvement. Upon examination he had a body temperature of 38.8 °C. His respiratory rate was 26 breaths/minute and oxygen saturation was 94% breathing room air. His heart rate was 98/min and blood pressure was 158/102 mmHg. Bilateral crackles were noted upon lung auscultation and his skin was icteric. Neurological status was normal. Laboratory blood tests revealed severe anemia with a hemoglobin (Hb) concentration of 80 g/L (reference interval 134–170 g/L), a leukemoid reaction with a total white blood cell count of 52.6 × 10^9/L (reference interval 3.5–8.8 ×10^9/L), with 64% neutrophils, and thrombocytosis with a platelet count of 824 × 10^9/L (reference interval 145–348 ×10^9/L). C-reactive protein in plasma (CRP) was 211 mg/L (reference interval >4 mg/L). Plasma creatinine was 127 μmol/L (reference interval 60–105 μmol/L), plasma sodium was 125 mmol/L (reference interval 137–145 mmol/L) and plasma potassium 4,3 mmol/L (reference interval 3.5–4.4 mmol/L). Liver enzymes, haptoglobin and lactate dehydrogenase (LDH) could not be analyzed due to hemolysis, but the total plasma bilirubin concentration was 87 μmol/L (reference interval 5–25 μmol/L), of which 9 μmol/L was conjugated, and INR was 1,0 (reference interval 0.9–1.2).

The patient was initially treated with piperacillin/tazobactam, intravenous fluids and ipratropium- and salbutamol inhalations. Computed tomography of the chest and bowel with intravenous contrast revealed signs of bilateral pneumonia but no abnormalities in the liver, biliary- or urinary tract. The second day hemoglobin decreased to 44 g/L and creatinine levels rose to 322 μmol/L. Direct antiglobulin test (DAT) was highly positive (4/4) for complement C3d and negative for IgG and showed a high titer of cold-agglutinin antibodies of > 640. Plasma haptoglobin was below the detection limit of 0,1 g/L (reference interval 0.24–1.90 g/L). Corticosteroid treatment was initiated with 16 mg betamethasone day one, followed by 8 mg daily doses against cold agglutinin hemolysis. The patient was transferred to a hematology unit at the university hospital where he received warm blood transfusions to prevent further cold hemolysis. He also received treatment with two doses of the erythropoietin analog darbepoetin. The antimicrobial therapy was complemented with Levofloxacin in reduced dose due to renal failure.

An extensive microbiological workup was performed. Polymerase chain reaction (PCR) from a pharynx swab was positive for *M. pneumoniae*. Bacterial cultures from blood, nasopharynx and urine were negative. PCR for *Legionella pneumophila, Chlamydophila psittaci* and *Chlamydophila pneumoniae* were negative as well as *Legionella pneumophila* and *Streptococcus pneumoniae* antigens in urine. Hepatitis A, B and C serology, Cytomegalovirus and Epstein-Barr virus serology, PCR for influenza virus, respiratory syncytial virus and metapneumovirus as well as Human Immunodeficiency virus (HIV) antigen- and antibody tests were negative. An immunologic workup showed no presence of anti-neutrophil cytoplasmic antibodies (ANCA) or anti-nuclear antibodies (ANA). Complement function was normal but there were signs of consumption by the classical pathway.

The renal function worsened during the initial 10 days of admission. Creatinine levels peaked at 725 μmol/L at day 10 after admission and plasma urea peaked at 31.5 mmol/L (reference interval 3.5–8.2 mmol/L) at day 8, but the patient did not require hemodialysis. An ultrasound examination of the kidneys showed no hydronephrosis but a marked increase in renal cortex echogenicity with normal thickness. Urine test strip was highly positive for protein (4/4) and erythrocytes (3/4) and positive for glucose (1/4). Urinary sediment showed erythrocytes, leukocytes and hyaline cylinders. Around the time of the plasma creatinine peak the patient entered a polyuric phase with daily diuresis exceeding 10 L requiring substitution with intravenous fluids.

**Fig. 1:**
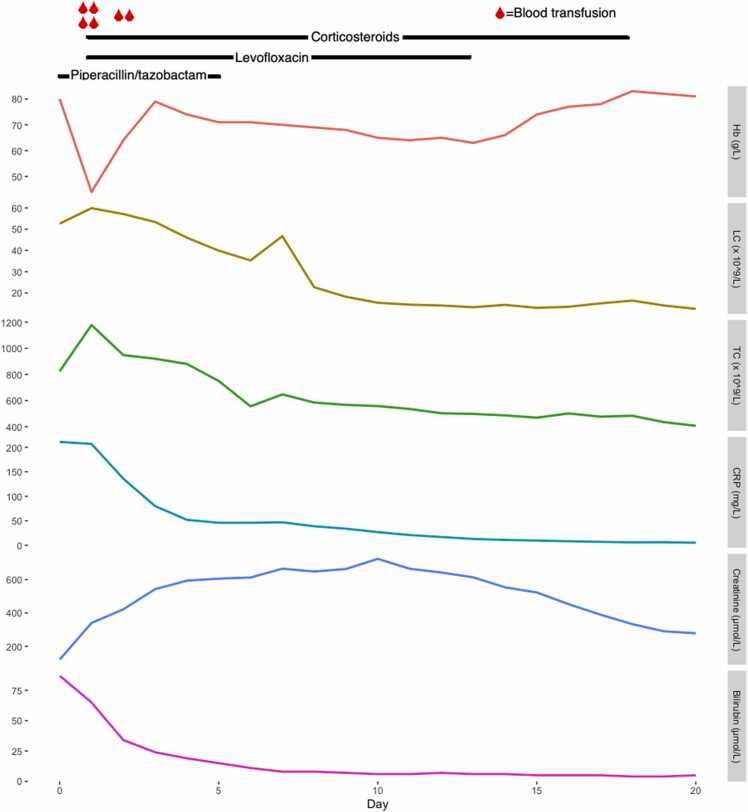
Development of laboratory parameters during hospital care. Hb=Blood hemoglobin, LC=Blood leukocyte count, TC=Blood thrombocyte count, CRP=Plasma CRP, Creatinine=Plasma creatinine, Bilirubin=Plasma bilirubin.

Treatment with piperacillin/tazobactam was discontinued after 5 days and Levofloxacin treatment was continued for an additional 7 days to a total of 12 days. The patient was afebrile after two days of treatment with levofloxacin and corticosteroids. Supplementary oxygen was administered intermittently but could be tapered after four days. Betamethasone treatment was switched to prednisolone in a single daily dose of 50 mg which was tapered over the following 12 days. Treatment was discontinued 17 days after initiation. Upon discharge plasma CRP had normalized (6.0 mg/L). Blood cell counts gradually improved, and upon discharge Hb was 81 g/L, leukocyte count 12.5 × 10^9/L and thrombocyte count was 407 × 10^9/L. Plasma creatinine decreased after day 10 and was 278 μmol/L upon discharge. The development of central laboratory parameters is shown in Fig. 1. At a three-month follow-up the patient was fully recovered, laboratory work-up and chest x-ray were normal.

## Discussion

*M. pneumoniae*-infection complicated by severe hemolytic anemia, renal function impairment or extensive leukocytosis are rare events that have previously been described separately. To our knowledge, this case is the first to report all three complications in the same patient.

*M. pneumoniae*-associated hemolysis is caused by cold agglutinin antibodies directed against the I antigen. This is a carbohydrate antigen present both on erythrocytes and respiratory epithelium cells, where it functions as the receptor mediating mycoplasma infection. The formation of antibodies against the I antigen is likely triggered by the interaction between *Mycoplasma* and the receptor, where the microbe acts as an adjuvant [7]. Antibodies against Sia-b1 on erythrocytes has also been reported and presence of antibodies directed at both these epitopes have been suggested to be highly specific for *M. pneumoniae* infection [8]. The binding of antibodies to erythrocytes is triggered by a lower temperature in the extremities and causes complement mediated hemolysis. Cold agglutinin antibodies have been observed in 50–60% of *M. pneumoniae* infections and evidence of subclinical hemolysis with elevated reticulocyte levels has been observed in the majority of M. pneumoniae cases [9], [10], [11]. Severe hemolysis though, is very rare, but ours and other described cases demonstrate that life-threatening anemia can develop quickly [12], [13], [14], [15], [16], [17]. The reason why high levels of cold-agglutinin antibodies and severe hemolysis emerge in some individuals is unknown. Our patient was under treatment with Methotrexate which in rare cases has been reported to cause immune hemolytic anemia and could theoretically have contributed to the dramatic course in this case [18].

No treatment studies of *M. pneumoniae* induced hemolysis have been carried out and optimal therapy is therefore unknown. Generally, focus should be on keeping the patient warm, and if blood transfusions are needed, these should be given with a blood warmer.

Glucocorticoid treatment has been shown to be beneficial in treatment of warm autoimmune hemolysis but its role in treatment of cold agglutinin hemolysis is not established [19]. In nine well described cases of *M. pneumoniae-*associated hemolysis, four patients received glucocorticoid treatment which coincided with a marked improvement in two of these cases [20]. Signs of reduced hemolysis were present in our patient on day three after glucocorticoid treatment-initiation with a steady decline of LDH, and haptoglobin normalized within a week. In cases where the hemolysis worsens despite supportive therapy and antibiotics it seems reasonable to administer glucocorticoids. Glucocorticoid therapy in *M. pneumoniae-*pneumonia without hemolysis is sometimes advocated but beneficial effects have not been well established and its use remains controversial [21], [22].

Antibiotics is generally recommended but probably play a limited role in the treatment of hemolysis. Theoretically, a faster clearance of the pathogen by antibiotic treatment could limit the production of cold agglutinins and their hemolytic effect.

In this case the patient also presented with a leukemoid reaction. Infections are the most common cause of leukemoid reactions and have been reported in association with a wide spectrum of infections such as *Clostridium dificile* (*C. dificile*)*, Mycobacterium tuberculosis* and *Shigella dysenterica*. Non-infectious causes include malignancies, intoxications, drug-reactions as well as bleeding and hemolysis [23]. *M. pneumoniae* pneumonia usually does not cause marked leukocytosis. And leukocytosis usually indicates another bacterial etiology than *M. pneumoniae* in pneumonia [24]*.* Regardless of cause, a leukemoid reaction is associated with a worsened prognosis with a high mortality [25] and it is a well-known negative prognostic marker in *C. difficile* infection [26]. Hemolysis has also been reported as a cause of leukemoid reactions and in this case it is hard to conclude whether it was a direct consequence of the infection itself or a response to hemolysis. We are aware of one more case-report were a leukemoid reaction was found in conjunction with severe cold agglutinin hemolysis in *M. pneumoniae* infection [15], which implies that the manifestations could be linked, although this remains speculative.

Our patient developed a marked thrombocytosis. More frequently, thrombocytopenia has been reported in association with *M. pneumoniae* infection, in some cases leading to fatal complications [27]. This illustrates that infection with *M. pneumoniae* should be considered in patients with pneumonia displaying hematological abnormalities.

Organ dysfunction such as renal failure, in association with a bacterial pneumonia may lead the clinician into suspecting a more virulent pathogen such as *Streptococcus pneumoniae* and initiate treatment with antibiotics that are not effective against *M. pneumoniae.* Our patient and other reported cases demonstrate that *M. pneumoniae* should be a differential diagnosis in patients with pneumonia and renal failure, especially if there are signs of hemolysis or other extra-pulmonary manifestations consistent with *M. pneumonia* infection. Whether the renal failure in this case was a direct consequence of the infection or caused by hemolysis in combination with the use of intravenous iodine contrast during computed tomography examination is hard to conclude. Nevertheless, it illustrates the importance of monitoring kidney function in patients with severe *Mycoplasma* infection.

## Ethical approval

Ethical approval is not required for case reports in our institution.

## Funding

This research did not receive any specific grant from funding agencies in the public, commercial, or not-for-profit sectors.

## CRediT authorship contribution statement

**Johan Widén:** data collection, manuscript preparation, literature review, involved in patient care, creation of figure. **Göran Jönsson:** involved in patient care, manuscript preparation. **Ulf Karlsson:** manuscript preparation.

## Conflicts of interest

The authors have no conflicts of interest to declare.
